# Do Canadians have favourable attitudes towards reintroducing mask mandates?

**DOI:** 10.17269/s41997-023-00805-1

**Published:** 2023-08-11

**Authors:** Frédérique Deslauriers, Camille Léger, Simon L. Bacon, Kim L. Lavoie, on behalf of the iCARE Study team

**Affiliations:** 1 Department of Psychology, Université du Québec à Montréal (UQAM), Montréal, QC Canada; 2 Montreal Behavioural Medicine Centre, Centre Intégré Universitaire de Santé et de Services Sociaux du Nord-de-l’Île-de-Montréal (CIUSSS-NIM), Montréal, QC Canada; 3 Department of Health, Kinesiology and Applied Physiology, Concordia University, Montréal, QC Canada

Dear Editor:

Like many countries, Canada faced a triple threat of respiratory illness (COVID-19, influenza, and respiratory syncytial virus) in the fall of 2022, after all COVID-19 measures had been lifted, placing significant pressure on the healthcare system (Tanne, 2022; Wright, 2022). Public health agencies were therefore urging the population to maintain preventive behaviours like masking indoors to help slow the spread of these viruses (Wright, 2022). Mask-wearing has been associated with a lower incidence of COVID-19 and has been shown to prevent the spread of respiratory viruses in general, including COVID-19 (Aravindakshan et al., 2022; Howard et al., 2021; Talic et al., 2021). Although governments recommended mask-wearing indoors, no mandates were reintroduced (Canadian Institute for Health Information, (n.d.); Wright, 2022). This may have been influenced by the perception that the population would reject the mandate, or worse, that it might trigger mass protests (Gaviola, 2022; Montpetit & MacFarlane, 2020). To optimize our COVID-19 responses and future pandemic preparedness, it would be important to determine the population’s attitudes towards the reintroduction of mask mandates under conditions of heightened risk, and their socio-demographic predictors.

The International COVID-19 Awareness and Responses Evaluation (iCARE) Study is an ongoing multi-wave cross-sectional survey of population attitudes and behaviours surrounding COVID-19 (Bacon et al., 2021). Analyses of 3015 age-, sex- and province-weighted Canadian adults recruited via Leger’s© online panel between September 5 and 12, 2022 show that the majority of Canadians (70%) would be in favour of reintroducing indoor mask mandates if the pandemic situation worsened. The strongest predictors of positive attitudes were being fully vaccinated or boosted. Being employed was associated with negative attitudes towards mask mandates, potentially due to concerns about needing to wear masks all day at work (Montpetit & MacFarlane, 2020). Those who had recovered from a previous COVID-19 infection were also less likely to be in favour of mask mandates, unlike those who were infected and still having symptoms. This suggests that recovery from infection may reduce risk perceptions and decrease motivation for wearing masks (Rosenstock, 1974). Though survey methods may be subject to some bias, respondents were well distributed across sociodemographic characteristics.

Findings have implications for government policy planning. Public health measures should be determined based on the pandemic situation and *evidence* of acceptability, not perceptions of these. This study provides evidence that the Canadian population would accept the reintroduction of indoor mask mandates if the pandemic situation worsened. Results indicating negative attitudes towards mask mandates among workers suggest a need to adapt indoor masking policies to increase acceptability, for example, only being required in high exposure and transmission risk workplaces (e.g., service sector, education). Further, risk assessment education may be needed for those who may be underestimating their risk of contracting COVID-19 after recovering from previous infections.

## Data Availability

The iCARE study data are available on request via the process identified here: https://mbmc-cmcm.ca/covid19/apl/2023.

## Code availability

All analysis was performed in SAS version 9.4. The codes are available on request by contacting the corresponding authors.
